# Supplementary material for: Immunomodulatory and pro-oncologic effects of ketamine and isoflurane anesthetics in a murine model
Source: PLoS One. 2023 Oct 10;18(10):e0292492. doi: 10.1371/journal.pone.0292492 (PMC10564181; doi:10.1371/journal.pone.0292492)
Supplement: Attachment — Submitted filename: PLOS ONE response to reviewers.docx [file pone.0292492.s003.docx]

This manuscript was previously submitted (PONE-D-23-07692) but timed out and was automatically withdrawn as we had not responded to an inquiry in time. Please find below a copy of that inquiry and our response. Thanks.

**Inquiry**:

Dear Dr. Muncey,

We've checked your submission and before we can proceed, we need you to address the following issues:

Thank you for your attention to our requests. We noted that the study involves the growth of tumors in mice. While we understand that humane endpoints were used in the study and the mice were scarified at day 14, we would be grateful if you could also provide additional information on the tumor size. Available guidelines suggest that the recommended maximum tumor size for animals is between 2000 mm3 (e.g., mice) and 4000 mm3 (rats), although larger sizes may be conditionally acceptable based on other variables, such as species, tumor location, or study-specific ethics committee approval.

Before we continue to peer review, please provide in mm3 the maximum tumor size for each animal included in the study within the Results section.

We've returned your manuscript to your account. Please resolve these issues and resubmit your manuscript within 21 days. If you need more time, please email the journal office at [plosone@plos.org](mailto:plosone@plos.org). We are happy to grant extensions of up to one month past this due date. If we don't hear from you within 21 days, we will withdraw your manuscript.

**Response**:

Since these tumors developed in the lungs, we could not use a size endpoint like a typical orthotopic or subcutaneous tumor. We monitored the mice for pulmonary issues, such as labored breathing, hunched posture, or >20%  weight loss. Below is an excerpt from the protocol for our specific experimental endpoint:

“Mice show signs of disseminated disease such as pulmonary metastasis indicated by difficult labored breathing. Mice will be observed for specific clinical signs of discomfort (e.g., failure to groom, inactivity, failure to respond to stimuli, isolation from cage mates, shivering, ataxia, shallow, rapid and/or labored breathing, pale mucous membranes, cyanosis, soiled anogenital area, vocalization, head tilt, circling, lack of inquisitiveness, and/or a hunched posture).”
